# The qEEG Signature of Selective NMDA NR2B Negative Allosteric Modulators; A Potential Translational Biomarker for Drug Development

**DOI:** 10.1371/journal.pone.0152729

**Published:** 2016-04-01

**Authors:** Deborah Keavy, Linda J. Bristow, Digavalli V. Sivarao, Margaret Batchelder, Dalton King, Srinivasan Thangathirupathy, John E. Macor, Michael R. Weed

**Affiliations:** 1 Genetically Defined Diseases and Genomics, Bristol-Myers Squibb Company, Wallingford, CT, United States of America; 2 Department of Veterinary Sciences, Bristol-Myers Squibb Company, Wallingford, CT, United States of America; 3 Discovery Chemistry, Bristol-Myers Squibb Company, Wallingford, CT, United States of America; The Scripps Research Institute, UNITED STATES

## Abstract

The antidepressant activity of the N-methyl-D-aspartate (NMDA) receptor channel blocker, ketamine, has led to the investigation of negative allosteric modulators (NAMs) selective for the NR2B receptor subtype. The clinical development of NR2B NAMs would benefit from a translational pharmacodynamic biomarker that demonstrates brain penetration and functional inhibition of NR2B receptors in preclinical species and humans. Quantitative electroencephalography (qEEG) is a translational measure that can be used to demonstrate pharmacodynamic effects across species. NMDA receptor channel blockers, such as ketamine and phencyclidine, increase the EEG gamma power band, which has been used as a pharmacodynamic biomarker in the development of NMDA receptor antagonists. However, detailed qEEG studies with ketamine or NR2B NAMs are lacking in nonhuman primates. The aim of the present study was to determine the effects on the qEEG power spectra of the NR2B NAMs traxoprodil (CP-101,606) and BMT-108908 in nonhuman primates, and to compare them to the NMDA receptor channel blockers, ketamine and lanicemine. Cynomolgus monkeys were surgically implanted with EEG radio-telemetry transmitters, and qEEG was measured after vehicle or drug administration. The relative power for a number of frequency bands was determined. Ketamine and lanicemine increased relative gamma power, whereas the NR2B NAMs traxoprodil and BMT-108908 had no effect. Robust decreases in beta power were elicited by ketamine, traxoprodil and BMT-108908; and these agents also produced decreases in alpha power and increases in delta power at the doses tested. These results suggest that measurement of power spectra in the beta and delta bands may represent a translational pharmacodynamic biomarker to demonstrate functional effects of NR2B NAMs. The results of these studies may help guide the selection of qEEG measures that can be incorporated into early clinical evaluation of NR2B NAMs in healthy humans.

## Introduction

Major Depressive Disorder (MDD) is a heterogeneous condition characterized by the core symptoms of pervasive, sustained, low mood and/or loss of interest in the environment accompanied by a constellation of other symptoms involving alterations in sleep, appetite, energy level, psychomotor function and cognition [1]. The monoamine system has been the focus of MDD research for many years and although numerous antidepressant drugs of this class are available, MDD symptoms are poorly treated in most patients. Limitations of monoaminergic antidepressants include a delayed onset of action and a large population who show either modest or no treatment response (30–53%) [2]. Therefore there is a high demand for novel MDD therapeutics. In recent years, the glutaminergic system, including N-methyl-D-aspartate (NMDA) receptors, has been an area of research interest in the neurobiology of MDD [3, 4]. Berman *et al*., (2000) were the first to publish a small (n = 7) novel, proof-of-concept clinical trial showing the non-selective NMDA receptor antagonist ketamine had rapid anti-depressant effects in patients with treatment-resistant depression (TRD) [5]. Zarate *et al*., (2006) confirmed these findings by demonstrating the robust, rapid (within 2 hrs) and sustained (more than 1 week) anti-depressant effects of a single dose of ketamine (0.5 mg/kg, i.v. infusion for 40 min) in TRD patients [6].

Several potential issues are associated with non-selective NMDA receptor channel blockers such as ketamine, including psychotomimetic or dissociative effects, cognitive impairment and abuse potential [7, 8]. However, it has been suggested that directly targeting the NMDA receptor complex by using low-affinity receptor antagonists or by modulating NMDA receptor subtypes may bring about rapid antidepressant effects with reduced adverse effects [4]. Proof of concept for these approaches has been achieved in the clinic with lanicemine (AZD-6765), a low-trapping NMDA channel blocker and traxoprodil (CP-101,606) a negative allosteric modulator (NAM) which selectively inhibits the NR2B subtype of NMDA receptors. In preliminary clinical trials, lanicemine showed antidepressant efficacy with a reduced side-effect profile relative to ketamine [9]. However, in a larger trial of antidepressant efficacy, lanicemine did not differ from placebo although the agent was still well tolerated [10]. IV infusion of traxoprodil also exhibited ketamine-like efficacy in TRD patients with the advantage of reduced adverse effects [11].

Sanacora et al., (2013) demonstrated the utility of qEEG as a clinical biomarker to demonstrate pharmacodynamic effects of ketamine and lanicemine [12]. Both ketamine and lanicemine modulate gamma power band of the qEEG in rats and humans and the plasma concentrations affecting qEEG measures were similar to those demonstrating antidepressant efficacy. For lanicemine, the qEEG biomarker was useful in confirming the pharmacodynamic effects at NMDA receptors and guiding dose selection. In addition to dose selection, pharmacodynamic biomarkers aid interpretation of negative results. For instance, should a clinical dose have no therapeutic effect and also no effect on the pharmacodynamic biomarker, it’s clear that the therapeutic hypothesis has not been tested. On the other hand, if a clinical dose has the anticipated effect on the pharmacodynamic biomarker and still has no therapeutic effect, this suggests that the mechanism was adequately tested but found to be ineffective.

**Fig 1:**
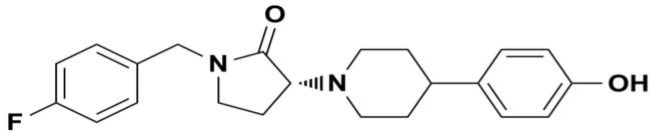
Structure of BMT-108908.

Subanesthetic doses of NMDA receptor channel blockers such as ketamine, phencyclidine or MK-801 produce very similar changes in qEEG measures in rodents and humans, whether in basal power spectra, or evoked potential procedures [13, 14]. The effects of NMDA antagonists on evoked potentials typically align well with rodents, nonhuman primates and humans, an indication of cross-species translatability of these measures [15–19]. However, there is a paucity of data describing the effects of NMDA antagonists and in particular, ketamine, on qEEG power spectra in nonhuman primates. Most early evaluations of ketamine used high doses in the anesthetic range [20]. In addition, most early reports did not analyze power spectra across a number of clinically relevant power bands. Similarly, there are few reports of the qEEG effects of NR2B selective NAMs in rodents [13, 14], and none to date in nonhuman primates. The primary goal of these studies was to determine if NR2B NAMs produce changes in qEEG spectra that may be useful as pharmacodynamic biomarkers. Therefore, the qEEG effects of two selective NMDA NR2B NAMs, traxoprodil and BMT-108908 (Fig 1) were studied in cynomolgus monkeys. To provide further insight into the qEEG effects of NMDA receptor channel blockers, ketamine and lanicemine were also examined. The results of these studies may help guide the selection of qEEG measures for pharmacodynamic biomarker studies in humans as well as provide insight into the effects of different classes of NMDA receptor antagonists in vivo.

## Materials and Methods

### Subjects

Six cynomolgus monkeys (Macaca Fasicularis) ~ 5–7 years of age and weighing 5.0–8.5 kg served as subjects for the qEEG studies. Each had been used previously in pharmacological studies, although there was a drug-free period of at least one month prior to these studies. Monkeys were typically pair- housed with compatible conspecifics, but separated during the work day (Monday—Friday) and re-paired after afternoon feeding. Monkeys were housed in standard stainless-steel macaque cages either 83x71x83cm or 79x79x83cm (W x L x H) with a pair sharing two adjoining cages. Colony rooms housed 12–16 cynos, and the monkeys remained in the colony room except for weekly EEG test sessions. Subjects were fed standard monkey chow (Harlan Teklad Global 20% protein Primate Diet 2050). Water was continuously available except during qEEG testing and fresh fruit was provided twice weekly. Toys and foraging devices were routinely provided and television programs were available in the colony rooms. Subjects were fitted with metal neck collars (Primate Products, Immokalee, FL). Laboratory animal care was according to Public Health Service Policy on the Humane Care and Use of Laboratory Animals, and Guide for the Care and use of Laboratory Animals, (2011). The protocol was approved by the Animal Care and Use Committee of the Bristol-Myers Squibb Company.

Subjects were surgically implanted under isoflurane anesthesia (2–4%) with radio-telemetry transmitters (Konigsberg Instruments, Inc., Monrovia, CA). Vital signs were monitored throughout the surgery (e.g. heart rate, expired CO2 percentage, pulse oxygenation, body temperature, etc) to ensure appropriate anesthesia and the health of the animal. The transmitters were attached to two pairs of EEG leads placed over the dura: one set with leads over the frontal cortex (with a reference over parietal cortex; roughly F6-P6 in the 10–20 system) and the second set with leads over the auditory cortex (roughly C6-CP6 in the 10–20 system). Buprenophine (0.01–0.03 mg/kg) was administered IM post surgery and then continued BID for 2–3 days. Animals were observed daily and analgesia was extended as needed if the animal exhibited signs of discomfort or as needed. After full recovery (approximately 3–4 weeks), the implanted radio-telemetry device was tested to ensure a good EEG signal, including auditory evoked potentials to 1 ms click evidenced over both derivations (data not shown). The data for this study was collected from the F6-P6 derivation for 5 monkeys. Artifact in the F6-P6 derivation of one monkey forced use of the C6-CP6 derivation. The patterns of qEEG changes did not differ from the C6-CP6 monkey and the other monkeys (data not shown).

### qEEG recording sessions

Animals were comfortably seated in a primate chair and acclimated to a quiet testing chamber outfitted with an antenna to receive telemeter signals and a camera to monitor the animal during the recording session. qEEG recording sessions included either intramuscular (IM) or intravenous (IV) drug administration. For IM studies, animals were placed in the test chamber, and after a 20 min baseline recording, animals were treated with either vehicle or drug and the qEEG measured for a further 90 min. For IV studies, prior to the start of qEEG baseline recordings, a catheter was placed in the saphenous vein and taped securely. After the baseline recording, the treatment (drug or vehicle) was administered and the catheter flushed with saline. The catheter was removed at the end of the test session. Animals were typically tested once weekly.

### qEEG data analysis

Integrated Telemetry Systems receivers processed analog signals from the telemeters and their analog output was digitized at 1,000 Hz by Powerlab DAQ systems using LabChart v7 (ADInstruments, Colorado Springs, CO). Raw EEG waveforms were Fourier transformed using a Han (Cosine Bell) window with 50% overlap between data blocks and an FFT block size of 1024. Overall total power (0.5–55 Hz), as well as power within different frequency bands was recorded for delta (0.5–4 Hz), theta (4–9 Hz), alpha (9–13 Hz), beta 1 (13–19 Hz), beta 2 (20–30 Hz) and gamma (30–55 Hz) bandwidths.

**Fig 2:**
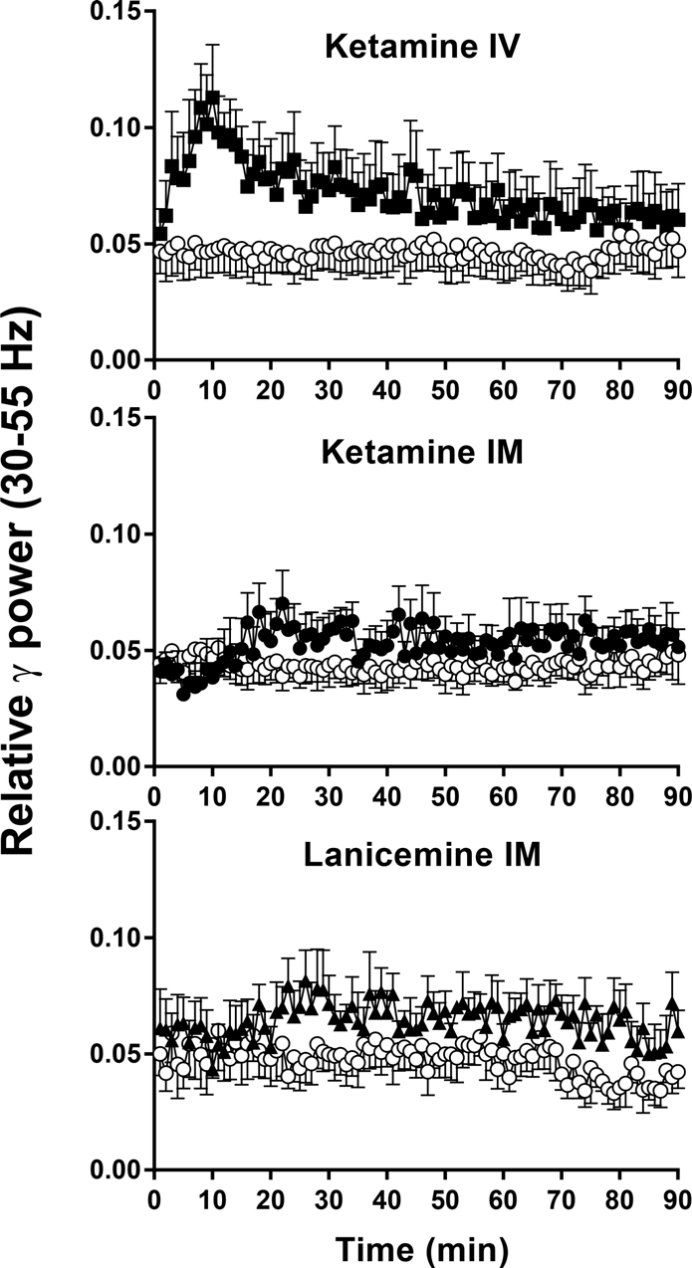
Non-selective NMDA channel blockers elicit robust changes in gamma (30–55 Hz) qEEG in cynomolgus monkeys. Y-axis is relative power in gamma (30–55 Hz) frequency band of the EEG power spectrum. X-axis is time after IM or IV administration. Results are the mean ± SEM (N = 5–6). Gamma band after 0.56 mg/kg IV ketamine (solid symbol) differs from vehicle (open symbol) at *p* < .01 level (Table 1). IM Ketamine (solid symbol) caused an increase in gamma relative power after 3 mg/kg, but was not significantly different from vehicle (open symbol), *p*>.05 (Table 1). Lanicemine 5.6 mg/kg IM (solid symbol), increases gamma band relative power and differs from vehicle (open symbol) at *p* < .05 level (Table 1).

**Fig 3:**
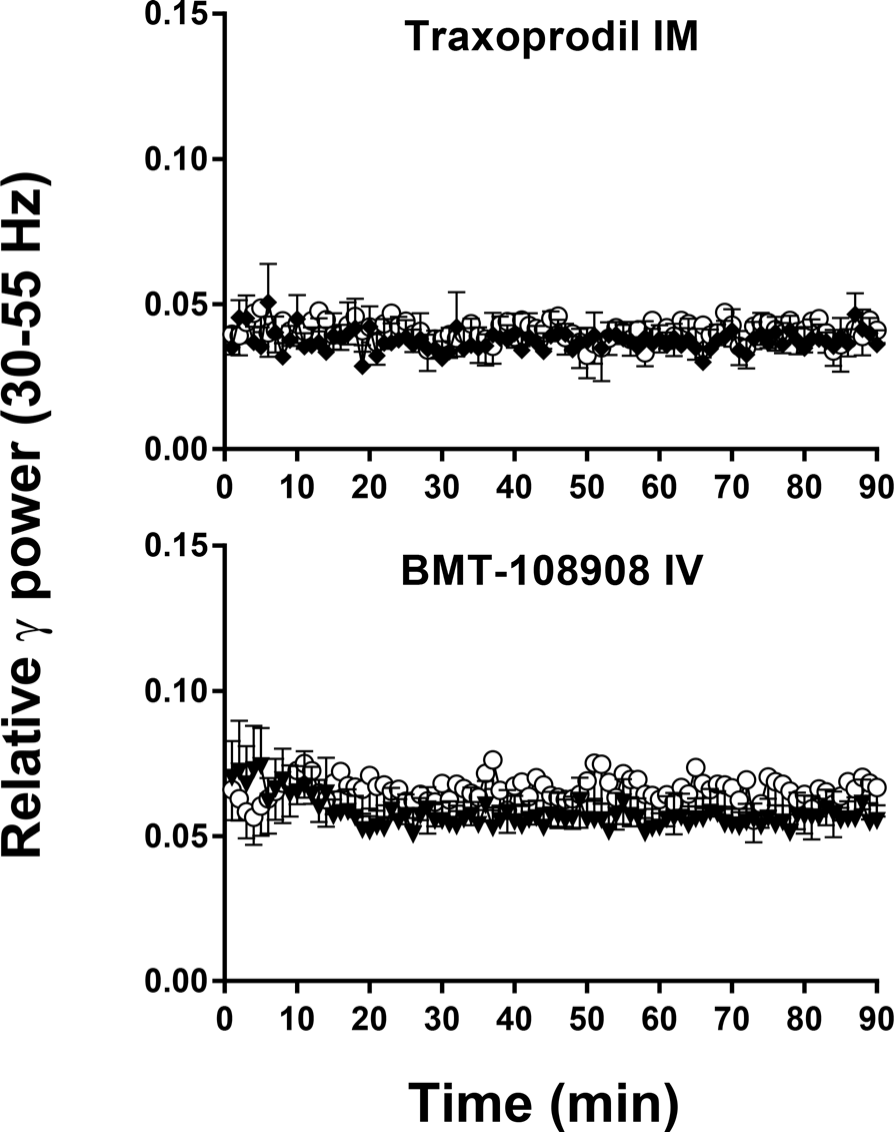
NR2b selective NAM’s have no effect on gamma (30–55 Hz) qEEG in cynomolgus monkeys. Y-axis is relative power in gamma (30–55 Hz) frequency band of the EEG power spectrum. X-axis is time after IM or IV administration. Results are the mean ± SEM (N = 5–6). Traxoprodil, 10 mg/kg IM and BMT-108908 3 mg/kg IV (closed symbol) had no effect on gamma, *p*>.05 (Table 1).

**Fig 4:**
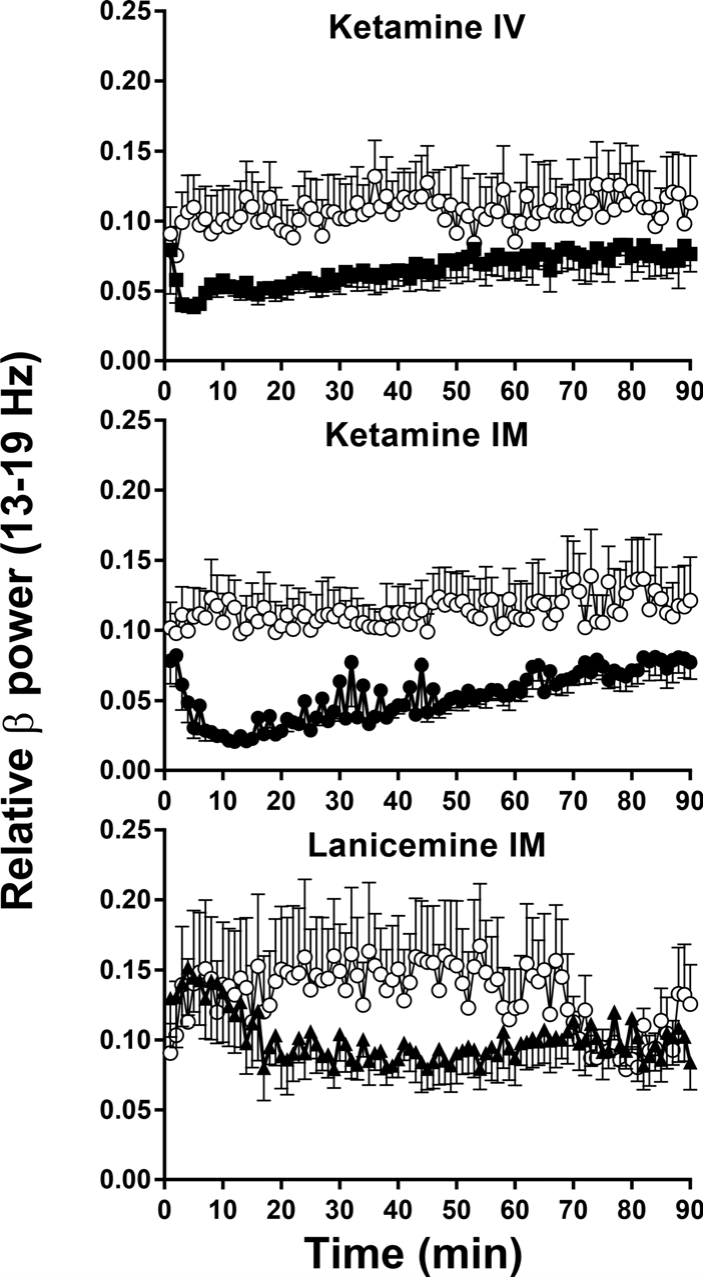
Non-selective NMDA channel blockers elicit robust changes in beta 1 (13–19 Hz) qEEG in cynomolgus monkeys. Y-axis is relative power in beta 1 (13–19 Hz) frequency band of the EEG power spectrum. X-axis is time after IM or IV administration. N = 5–6. Error Bars are SEM. Beta 1 band after 0.56 mg/kg IV, ketamine (closed symbol) differs from vehicle (open symbol) at p < .05 level. (Table 1). Ketamine caused a decrease in beta 1 relative power after 3 mg/kg IM (closed symbol), and differs from vehicle (open symbol) at p < .001 level (Table 1). Lanicemine 5.6 mg/kg IM (closed symbol), decreased beta 1 relative power but was not significantly different from vehicle (open symbol), p>.05 (Table 1).

**Fig 5:**
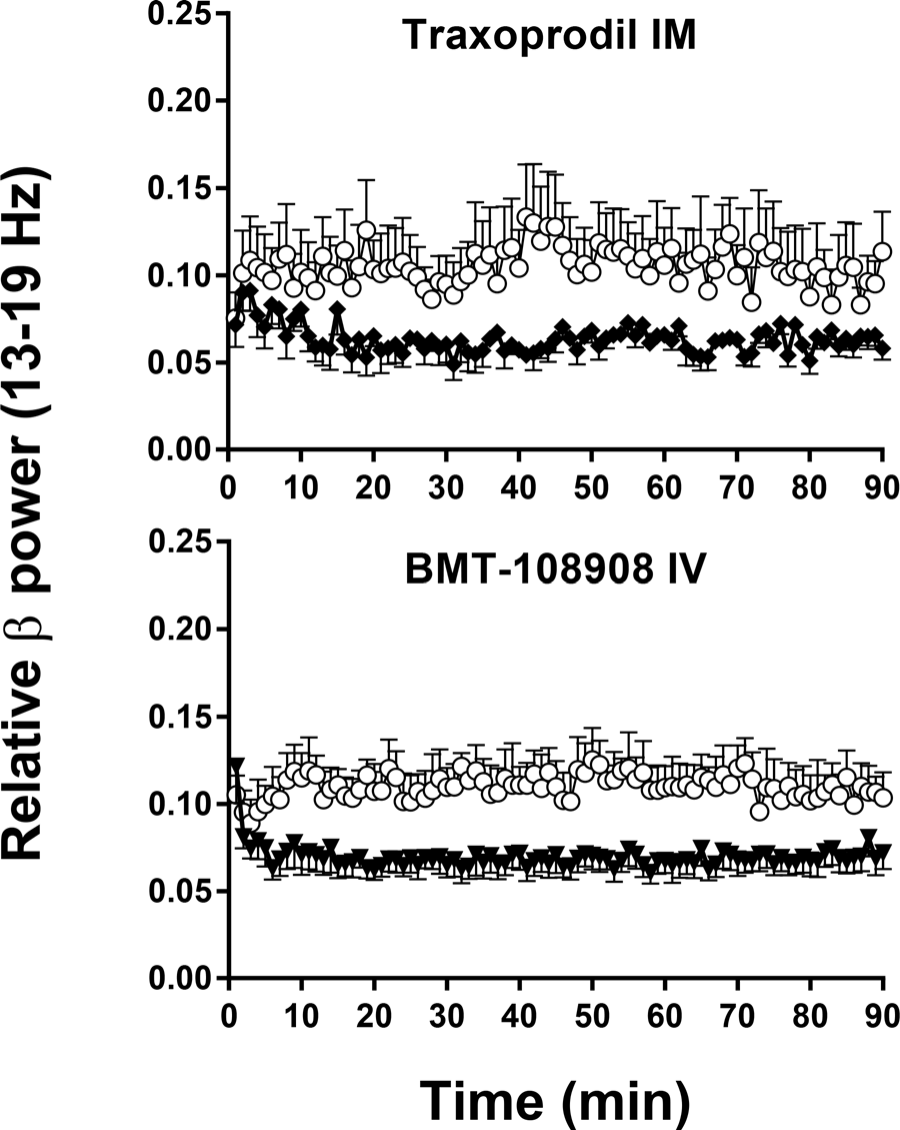
NR2b selective NAM’s elicit robust changes in beta 1 (13–19 Hz) qEEG in cynomolgus monkeys. Traxoprodil, 10 mg/kg IM (closed symbol) caused a reduction in beta 1 relative power and differs from vehicle (open symbol) at p < .05 level (Table 1). BMT-108908 3 mg/kg IV (closed symbol) decreased beta 1 band and differs from vehicle (open symbol) at p < .001 (Table 1).

The relative power for each frequency band (power in a band / overall total power) was determined as the average value from one min bins across the 90 min session. These data are provided as supplementary information (S1 Data). Area under the curve (AUC) over the entire 90 min period for each power band was calculated from the time course data and analyzed by 1-way RM ANOVA followed by Holm-Sidak post-hoc tests comparing vehicle to each dose of compound. The Holm-Sidak method was used for post-hoc comparisons between individual drug doses and vehicle (Prism v. 6.0, Graphpad Software Inc., La Jolla CA). Visual inspection of the qEEG relative power time course plots revealed a slower onset of activity for lanicemine relative to other drugs tested (most prominent in Fig 2; also present in Figs 3–5). Therefore a 15 min ‘pretreatment time’ was imposed on the analysis of lanicemine’s effects and AUCs were calculated for 16–90 min, analyzed by RM ANOVA, and used in Cohen’s d estimates. Pretreatment times were not necessary for other compounds administered IM as effects were more rapid with ketamine and traxoprodil (e.g. Fig 5). Summary statistics for the main effect of each RM ANOVA are presented in Table 1. Significant post-hoc tests are reported in the text. Additionally, the Cohen’s d estimate of effect size was calculated from the AUCs using unpaired statistics so as not to overestimate the effect size using an online tool from Becker, 2000 (http://www.uccs.edu/~lbecker/). Cohen’s d values of >0.2, >0.5 and >0.8 are described using the conventions of small, medium, and large effects, respectively [21].

**Table 1:** Summary of RM ANOVAs on relative power AUC for treatment compared to vehicle.

EEG BAND	Ketamine IV	Ketamine IM	Lanicemine IM	Traxoprodil IM	BMT-108908 IV
GAMMA	**F _(2, 8)_ = 5.655;**	F _(4, 16)_ = 0.9568;	**F _(3, 12)_ = 3.730;**	F _(3, 12)_ = 1.956;	F _(3 15)_ = 1.229;
(30–55 Hz)	***p* = 0.0295**	*p* = 0.4575	***p* = 0.0419**	*p* = 0.8974	*p* = 0.3336
BETA 2	F _(2, 8)_ = 1.113;	F _(4, 16)_ = 1.843;	F _(3, 12)_ = 1.775;	**F _(3, 12)_ = 3.657;**	**F _(3 15)_ = 7.838;**
(20–30 Hz)	*p* = 0.3745	*p* = 0.1699	*p* = 0.2053	***p* = 0.0442**	***p* = 0.0022**
BETA 1	**F _(2, 8)_ = 4.880;**	**F _(4, 16)_ = 6.584;**	**F _(3, 12)_ = 6.218;**	**F _(3, 12)_ = 5.379;**	**F _(3 15)_ = 10.42;**
(13–19 Hz)	***p* = 0.0412**	***p* = 0.0025**	***p* = 0.0086**	***p* = 0.0140**	***p* = 0.0006**
ALPHA	F _(2, 8)_ = 2.607;	**F _(4, 16)_ = 5.701;**	F _(3, 12)_ = 1.320;	F _(3, 12)_ = 1.981;	**F _(3 15)_ = 3.954;**
(9–13 Hz)	*p* = 0.1344	***p* = 0.004**	*p* = 0.3136	*p* = 0.1707	***p* = 0.0292**
THETA	F _(2, 8)_ = 1.868;	F _(4, 16)_ = 0.6031;	F _(3, 12)_ = 1.870;	F _(3, 12)_ = 0.3632;	F _(3 15)_ = 1.146;
(4–9 Hz)	*p* = 0.2160	*p* = 0.6660	*p* = 0.1885	*p* = 0.7808	*p* = 0.3627
DELTA	F _(2, 8)_ = 0.205;	**F _(4, 16)_ = 3.528;**	F _(3, 12)_ = 1.444;	F _(3, 12)_ = 2.275;	**F _(3 15)_ = 4.502;**
(0.5–4 Hz)	*p* = 0.8185	***p* = 0.0302**	*p* = 0.2789	*p* = 0.1320	***p* = 0.0192**

Summary of one way RM ANOVA main effects for relative power AUCs after treatment. AUC is calculated as relative power change from baseline for each animal at each dose and compared to the respective vehicle using a 1-way RM ANOVA with the factor of treatment (dose). Main effects with p<0.05 in bold.

### Drugs

Racemic ketamine HCl was purchased as a 10 mg/kg solution (Ketaset, Fort Dodge Animal Health, Fort Dodge, IA). CP 101,606 (traxoprodil, methanesulfonic acid salt, vehicle: 5% DMSO/10% propylene glycol/15% methylcellulose/70% sterile water), AZD6765 (lanicemine, HCl, vehicle: 5% DMSO/95% sterile water), and BMT-108908 ((3R)-1-[(4-fluorophenyl)methyl]-3-[4-(4-hydroxyphenyl)piperidin-1-yl]pyrrolidin-2- one [22] (Fig 1) free base, vehicle: 30% hydroxypropyl beta cyclodextrin/70% citrate buffer, pH 4.0) were synthesized at Bristol-Myers Squibb. Compounds were administered either IM or IV at 0.4 ml/kg. Ketamine was administered both IM and IV to provide a positive control in both dosing conditions. All doses refer to the free base.

## Results

### Effect of IV ketamine on qEEG

**Fig 6:**
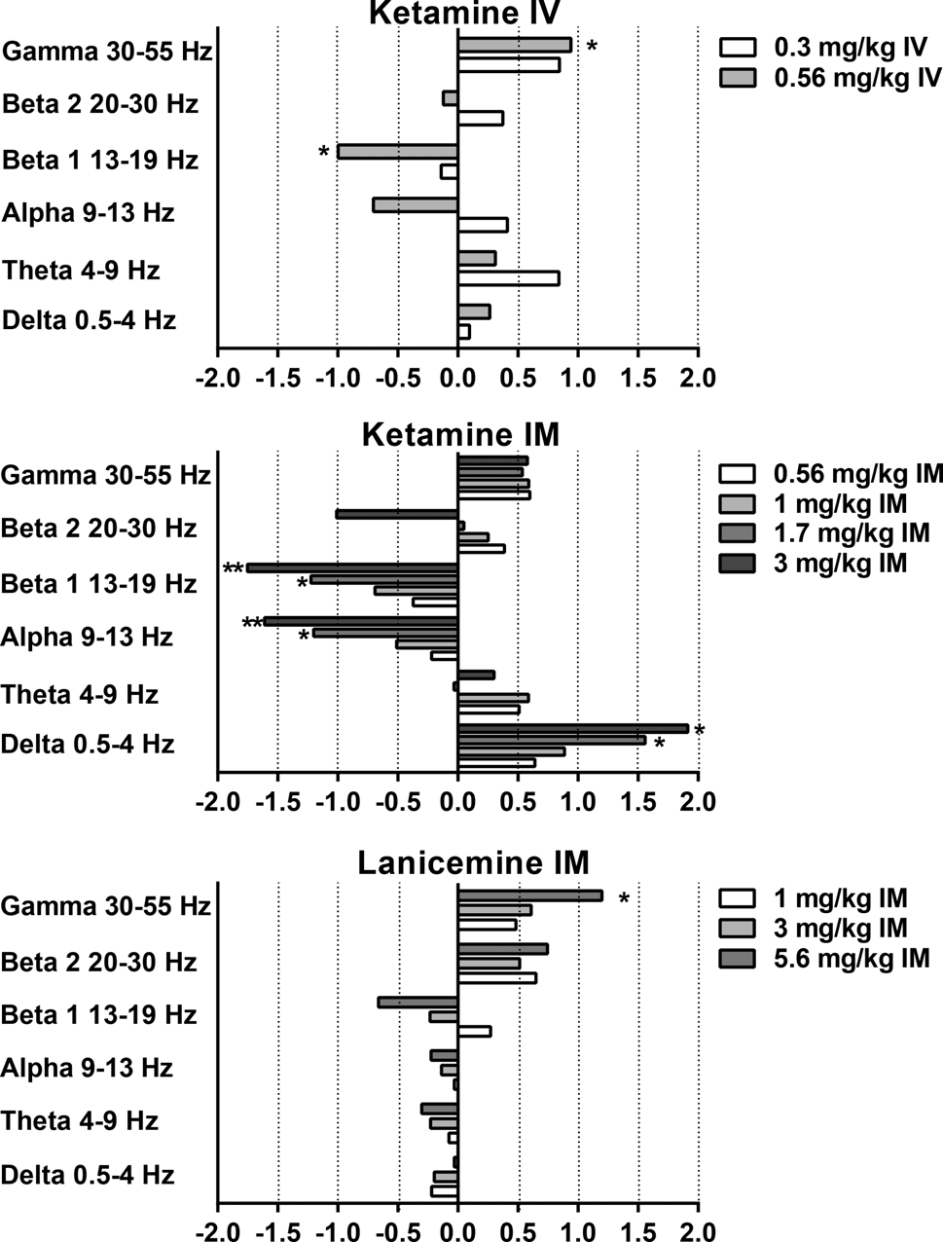
Non-selective NMDA channel blockers elicit robust changes in qEEG power spectra in cynomolgus monkeys. Y-axis is relative power in designated frequency bands of the qEEG power spectrum. X-axis is Cohen’s d’ calculated from the area under the relative power curve for each band. N = 5–6. Significant changes from qEEG AUC following vehicle are designated by *, **, or *** indicating significant differences at the *p* < .05, *p* < .01, and *p* < .001 levels, respectively.

As indicated in Table 1, IV ketamine produced statistically significant main effects of treatment on relative power in gamma and beta 1 bands. Post hoc tests confirmed a significant increase in gamma power after 0.56 mg/kg (*p* = 0.0216, Fig 2) with a Cohen’s d = 0.94 confirming a large effect (Fig 6). There was also a significant decrease in the beta 1 power band after 0.56 mg/kg (*p* = 0.0404, Fig 4) with a Cohen’s d = 1.00 confirming a large effect (Fig 6). IV ketamine had no significant effects on other power bands. Despite a medium Cohen’s d effect size of 0.7, the decreases in relative alpha power IV ketamine did not reach statistical significance (Holm-Sidak *p*> 0.05).

### Effect of IM ketamine on qEEG

As indicated in Table 1, IM ketamine produced statistically significant main effects of treatment on relative power in beta 1, alpha and delta bands. Similar to IV ketamine, IM ketamine increased gamma relative power (Fig 2) with Cohen’s d effect sizes of ~ 0.6 (Fig 6); however, these increases in gamma power did not reach significance following RM-ANOVA (*p>*0.05). Also similar to IV ketamine, IM ketamine produced significant dose dependent decreases in beta 1 (Fig 4) with post hoc tests confirming decrease after 1.7 mg/kg and 3 mg/kg (*p* = 0.0112 and *p =* 0.0011, respectively; Fig 6). Cohen’s d effect sizes for beta 1 decreases were large at 1.2 and 1.8 after 1.7 mg/kg and 3 mg/kg, respectively. Significant decreases were also seen in alpha relative power at these doses with large Cohen’s d effect sizes of 1.2 and 1.6 after 1.7 mg/kg and 3 mg/kg (post hoc *p* = 0.0205 and *p* = 0.0037, respectively). In contrast, delta power was significantly increased after 1.7 mg/kg and 3 mg/kg IM ketamine with Cohen’s d effect sizes of 1.6 and 1.9 (post hoc *p* = 0.0303 and *p* = 0.0214, 3 mg/kg, respectively). Beta 2 and theta bands did not significantly differ from vehicle (*p*>0.05).

### Effect of IM lanicemine on qEEG

As indicated in Table 1, IM lanicemine produced statistically significant main effects of treatment on relative power in gamma and beta 1 bands. Lanicemine produced a dose-dependent increase in gamma power with statistical significance after 5.6 mg/kg IM (*p* = 0.0186, Fig 2) with a Cohen’s d effect size of 1.20. Despite a significant main effect in the beta 1 band, and a medium Cohen’s d effect size of 0.66, the decrease in beta 1 following 5.6 mg/kg narrowly missed statistical significance on the Holm-Sidak post hoc test (p = 0.0578; Fig 4). Trends toward increases in beta 2 relative power were not significantly different from vehicle (*p*>0.05). Lanicemine also had no significant effect on alpha, delta or theta relative power at the doses tested (*p*>0.05).

### Effect of IM traxoprodil on qEEG

**Fig 7:**
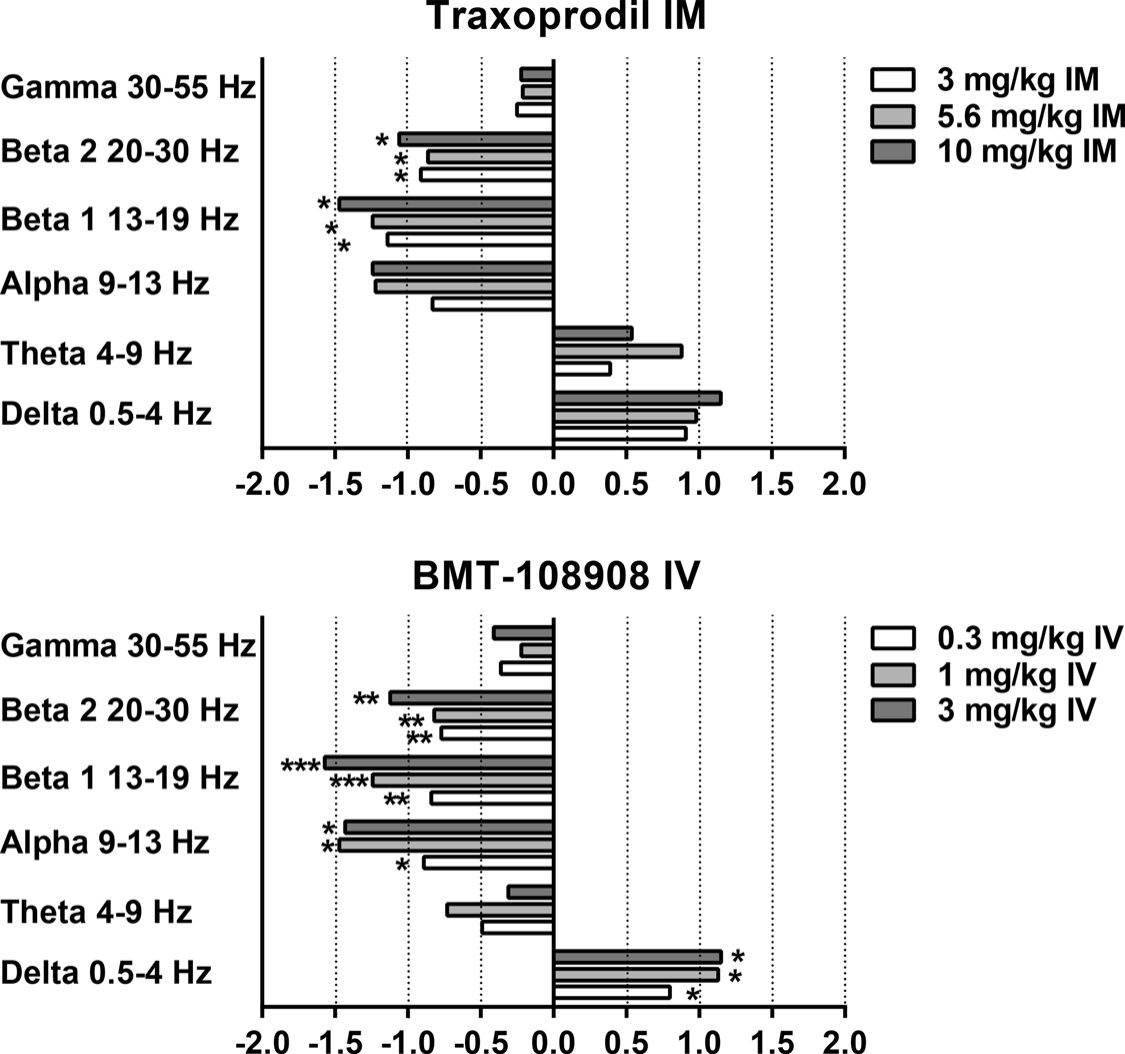
NR2b selective NAM’s elicit robust changes in qEEG power spectra in cynomolgus monkeys. Y-axis is relative power in designated frequency bands of the qEEG power spectrum. X-axis is Cohen’s d’ calculated from the area under the relative power curve for each band. N = 5–6. Significant changes from qEEG AUC following vehicle are designated by *, **, or *** indicating significant differences at the *p* < .05, *p* < .01, and *p* < .001 levels, respectively.

The NR2B NAM traxoprodil had no effect on gamma relative power (*p*>0.05, Fig 3). However statistically significant decreases were observed in beta 1 after 3.0, 5.6 mg/kg and 10 mg/kg IM (Fig 5) with large Cohen’s d effect sizes of 1.42, 1.10 and 1.23, respectively (*p* = 0.0141 for each; Fig 7). All doses of traxoprodil tested also produced, statistically significant decreases in beta 2 relative power with large Cohen’s d effect sizes of 0.91, 0.96 and 1.061 at 3.0, 5.6 mg/kg and 10 mg/kg, respectively (*p* = 0.0442 for each; Fig 7). Decreases in alpha power were not statistically significant despite large Cohen’s d effect sizes (0.83, 1.24 and 1.26 after 3.0, 5.6 and 10 mg/kg, respectively). Similarly, increases in relative power in the delta band were not statistically significant despite large Cohen’s d effect sizes (0.91, 0.98 and 1.152 after 3.0, 5.6 and 10 mg/kg, respectively). No significant effects on power in the theta band were observed.

### Effect of IV BMT-108908 on qEEG

The NR2B NAM BMT-108908 had no effect on gamma relative power (*p*>0.05, Fig 3). However, significant decreases were seen in beta 1 relative power at all doses tested (*p* = 0.0041, *p* = 0.0009 and *p* = 0.0004 for 0.3, 1.0, and 3.0 mg/kg respectively; Fig 5). Cohen’s d effect sizes were large at 0.8, 1.2 and 1.6 after 0.3, 1.0 and 3.0 mg/kg, respectively (Fig 7). Similarly, significant decreases were seen in beta 1 relative power at all doses tested (*p* = 0.00490, *p* = 0.0049, *p* = 0.0013 for 0.3, 1.0, and 3.0 mg/kg respectively) with Cohen’s d effect sizes of 0.77, 0.82 and 1.12, respectively. Significant decreases on alpha relative power were also seen with all doses (*p* = 0.0467 at 0.3 mg/kg, *p* = 0.0223 at 1 mg/kg and *p* = 0.0242 at 3 mg/kg) with Cohen’s d effect sizes of 0.89 at 0.3 mg/kg, 1.47 at 1 mg/kg and 1.43 at 3 mg/kg. BMT-108908 had no significant effect on relative theta power.

## Discussion

The present studies are the first to demonstrate the effect of NR2B selective NAMs as well as the NMDA receptor channel blockers ketamine and lanicemine, on quantitative EEG in nonhuman primates including a full range of clinically relevant frequency bands. In general, the channel blockers ketamine and lanicemine increased gamma power and decreased beta power. In contrast to the NMDA channel blockers, the selective NR2B NAMs traxoprodil and BMT-108908 had no effect on gamma power. However, the NR2B NAMs produced robust decreases in alpha, beta 1 and beta 2 frequency bands. Both ketamine and the NR2B NAMs increased power in the delta frequencies.

While human data for NR2B NAMs are lacking, the present data demonstrating no effect on gamma power following treatment with NR2B NAMs is consistent with previously published reports in rats [13, 14, 19]. The Kocsis et al., (2012) study provided evidence for subunit-specificity of cortical gamma activity induced by NMDA receptor blockade and showed that increases in aberrant gamma activity are primarily mediated by NMDA receptor antagonists containing the NR2A subunit, whereas effecting receptors containing NR2B, NR2C, or NR2D subunits did not produce this response [14]. Similarly, Sivarao et al., 2014 found no change in gamma and decreases in beta 1 and beta 2 bands after traxoprodil administration in rats [13]. Therefore, the lack of effect of traxoprodil and BMT-108908 on gamma power in the nonhuman primate is consistent with published reports in rats. The robust changes in beta, alpha and delta frequencies following NR2B NAM administration suggest that these frequency bands may be useful as pharmacodynamic biomarkers in future clinical studies.

The primary clinical route of administration for NMDA receptor channel blockers and NR2B NAMs has been intravenous; however, clinical evidence for antidepressant activity after intramuscular injection of ketamine is growing [23]. Therefore, both routes were included in these studies. Additionally one NR2B NAM was administered via each route. Ketamine is rapidly eliminated from plasma after IV administration [24], and its EEG effects in humans closely follow its pharmacokinetic profile [25]. The rapid rise and fall of gamma power after IV ketamine in the present study is consistent with its profile in humans [25]. However, when analyzed by area under the curve for the measure of effect size, the shorter profile after IV likely blunted ketamine’s qEEG effects relative to IM administration in the present study. Nonetheless, the overall pattern of effects across power bands is similar between IM and IV ketamine. The direction of power changes were also similar for the two NR2B NAMs whether given IM or IV; therefore, the route of administration does not appear to be a large factor in the effect of channel-blocking NMDA antagonists or NR2B NAMs on qEEG power spectra.

One limitation of the present studies was the relatively small number of subjects. While 5–6 macaques is similar in size to many nonhuman primate EEG studies previously published [15, 16, 18], relatively small variation between subjects can influence statistical significance of the analysis. For instance traxoprodil increased delta power with relatively large effects in all subjects; however, differences in the sensitivity of individual animals to different doses (e.g. largest effect at 3 mg/kg in one animal and at 10 mg/kg in another) introduced sufficient variability for the RM ANOVA to fail to reach significance. However, the level of technical difficulty and the resource-intensiveness of the model did not allow for additional subjects to be included.

Another potential concern with the methodology is that the comfortable, dimly-lit, environment of the sound-attenuating cubicle could possibly cause the monkeys to sleep. Decreases in higher frequency bands (e.g. gamma or beta) and increases in lower frequency power bands (e.g. delta) may be consistent with sleep induction; however, subjects were continuously monitored via video camera during each session and there were no observations of eye closure or sleep when decreased beta and increased delta power occurred (data not shown). In addition, similar doses of these agents impaired cognition in cynomolgus monkeys without producing signs of motor impairment or sedation during operant tests performed in similar cubicles [26]. Therefore, the qEEG effects of NR2B NAMs were not likely to be due to induction of sleep.

A reliable pharmacodynamic biomarker should show a systematic relationship with other pharmacologic effects of the compound. These same four compounds were all recently shown to impair cognition in a delayed-match to sample (DMS) test in cynomolgus monkeys [26]. Interestingly, all of the doses of compounds that produced significant qEEG effects also produced behavioral effects on the DMS task. While there is not a 1:1 correspondence between qEEG and DMS effects (e.g. lower ketamine doses of 0.56 and 1.0 mg/kg affected DMS but not qEEG) there is good agreement between doses changing qEEG power and doses impairing cognition. Overall, these results suggest that qEEG changes can be a useful measure of pharmacodynamic activity in the brain with either NMDA channel blocking antagonists or NR2B NAMs.

An important caveat for qEEG biomarkers is the possibility of biphasic qEEG responses. Indeed, doses of ketamine higher than those used here become anesthetic, and in both macaques and humans, power spectra are likely to differ once anesthetic effects are achieved. Accordingly increases in beta and theta power have been reported after anesthetic doses of ketamine in rhesus monkeys (10–15 mg/kg IM) and humans (approximately 4 mg/kg IV) [20, 25]. In the present context of developing novel antidepressants, it is unlikely that a biphasic response of qEEG would be relevant, as antidepressant doses are much lower than anesthetic doses of NMDA channel blocking antagonists.

In addition to aiding development of clinical biomarkers, the results of the present studies highlight something interesting about how cortical gamma oscillations may relate to antidepressant effects of NMDA antagonists. Acute ketamine administration in humans induces psychomimetic and dissociative effects coincident with an increase in gamma oscillations (whether measured by spontaneous qEEG or as gamma oscillations following evoked potentials) [12, 27]. It is not clear whether the increase in gamma power is a necessary component of the antidepressant effects produced by NMDA antagonists. However, results of preclinical studies, including this one, question that hypothesis. While NR2B NAMs show little increase in cortical gamma in rodents, they do have robust anti-depressant effects across species. This is the first report that establishes that as in rodents, NR2B NAMs do not affect gamma oscillations in primates. As mentioned previously, traxoprodil has shown antidepressent effects, however, EEG was not measured in those studies, and it is unknown how gamma oscillations were affected. In addition, Lanicemine, a low trapping NMDA channel blocker, was shown to produce robust increase in gamma oscillations in rodents, non-human primates (current report) as well as healthy human subjects [12]. However, despite the initial promise, that compound did not show therapeutic efficacy in large multi-center trials. Thus, there may be a double dissociation as far as gamma oscillations and efficacy in MDD is concerned: an increase in acute gamma oscillations does not ensure a long-term efficacy against depression (lanicemine) while a lack of gamma oscillations does not preclude NR2B agents from being effective in the clinic against MDD (traxoprodil). The use of qEEG as pharmacodynamic biomarkers for NR2B NAM antidepressants may also provide clinical data that would help understand the relationship between increases in gamma power antidepressant efficacy of NR2B antidepressants.

In summary, the qEEG effect profiles of traxoprodil and BMT-108908 in the non-human primate are strikingly similar with comparable effects seen across all power bands. These results suggest that, while elevations in gamma band power may provide evidence for NMDA channel block, changes in other qEEG bands may provide pharmacodynamic biomarkers for NR2B NAMs, specifically the reduction in beta and increase in delta power. The results of this study provide further evidence of the translation across species of qEEG measures and support its utility as a pharmacodynamic biomarker and suggest that qEEG models may represent a clinical biomarker for pharmacodynamic effect of NMDA antagonists and NR2B NAMs in early clinical trials.

## Supporting Information

S1 Data Relative power data spreadsheet file includes relative power in each band for each treatment for each subject. Relative power spectra are averages of power across one minute bins over the 90 min session for each individual subject. Data are normalized to that individual subject’s pre dose baseline levels for each spectral band. These are the data used to construct figures and analyzed to build the ANOVA table.
(XLSX)
